# Genetic Diversity and Population Structure of *Busseola segeta* Bowden (Lepidoptera; Noctuidae): A Case Study of Host Use Diversification in Guineo-Congolian Rainforest Relic Area, Kenya

**DOI:** 10.3390/insects3041156

**Published:** 2012-11-06

**Authors:** George O. Ong’amo, Bruno P. Le Ru, Pascal Campagne, Antoine Branca, Paul-Andre Calatayud, Claire Capdevielle-Dulac, Jean-Francois Silvain

**Affiliations:** 1 School of Biological Science, College of Physical and Biological Sciences (Chiromo Campus), University of Nairobi, Nairobi 30197, Kenya; 2 Unité de Recherche IRD 072, International Centre of Insect Physiology and Ecology (ICIPE), Nairobi 30772, Kenya; E-Mails: bleru@icipe.org (B.P.L.R.); pcalatayud@icipe.org (P.-A.C.); 3 Unité de Recherche IRD 072, International Centre of Insect Physiology and Ecology (ICIPE), Université Paris-Sud 11, Orsay cedex 91405, France; E-Mail: Pascal.Campagne@legs.cnrs-gif.fr; 4 Unité de Recherche IRD 072, CNRS, Laboratoire Evolution, Génomes et spéciation, UPR 9034, 91198 Gif-sur-Yvette cedex, France et Université Paris-Sud 11, Orsay cedex 91405, France; E-Mails: antoine.branca@legs.cnrs-gif.fr (A.B.); Claire.Capdevielle-Dulac@legs.cnrs-gif.fr (C.C.-D.); jean-francois.silvain@legs.cnrs-gif.fr (J.-F.S.)

**Keywords:** Cytochrome *b*, exchange, growing seasons, haplotypes, wild habitat, *Zea mays*

## Abstract

Habitat modification and fragmentation are considered as some of the factors that drive organism distribution and host use diversification. Indigenous African stem borer pests are thought to have diversified their host ranges to include maize [*Zea mays* L.] and sorghum [*Sorghum bicolor* (L.) Moench] in response to their increased availability through extensive cultivation. However, management efforts have been geared towards reducing pest populations in the cultivated fields with few attempts to understand possible evolution of "new" pest species. Recovery and growing persistence of *Busseola segeta* Bowden on maize (*Zea mays* L.) in Kakamega called for studies on the role of wild host plants on the invasion of crops by wild borer species. A two-year survey was carried out in a small agricultural landscape along the edge of Kakamega forest (Kenya) to assess host range and population genetic structure of *B*. *segeta.* The larvae of *B*. *segeta* were found on nine different plant species with the majority occurring on maize and sorghum. Of forty cytochrome *b* haplotypes identified, twenty-three occurred in both wild and cultivated habitats. The moths appear to fly long distances across the habitats with genetic analyses revealing weak differentiation between hosts in different habitats (*F*_ST_ = 0.016; *p* = 0.015). However, there was strong evidence of variation in genetic composition between growing seasons in the wild habitat (*F*_ST_ = 0.060; *p* < 0.001) with emergence or disappearance of haplotypes between habitats. *Busseola segeta* is an example of a phytophagous insect that utilizes plants with a human induced distribution range, maize, but does not show evidence of host race formation or reduction of gene flow among populations using different hosts. However, *B*. *segeta* is capable of becoming an important pest in the area and the current low densities may be attributed to the general low infestation levels and presence of a wide range of alternative hosts in the area.

## 1. Introduction

Natural ecosystems provide important habitats for a wide range of organisms. Unfortunately, these habitats have been subjected to diverse forms of modifications over the past half century resulting in significant loss and fragmentation of natural ecosystems [1,2,3,4]. Organisms exposed to these modifications, particularly phytophagous insects, exhibit a wide range of responses varying from host range expansion to local species extinction [5,6,7,8]. The ability of insects to utilize different host plants has been suggested to be a dynamic and transient phase [9]. During or after this phase, species can shift to novel host plants or re-specialize on ancestral ones. Expanding the range of host plants might also be a factor leading to higher net speciation rates. Lepidopteran stem borers are among the phytophagous insects that expanded their host ranges upon exposure to anthropogenic changes. In East Africa, stem borer pests, *Busseola fusca* Fuller and *Sesamia calamistis* Hampson, are examples of phytophagous insects that expanded their hosts and eventually specialised to feed on maize and sorghum where they remain important pests [10]. Though trade-offs associated with host use diversification have been evaluated in different contexts and applied in stem borer pest management (e.g., in the "Push-Pull" system, [11]), this study focused on the effects of fine scale host use diversification on genetic structure of a native phytophagous stem borer sub-species, *Busseola segeta *Bowden, and potential influence of natural habitats on its dynamics.

*Busseola segeta* was for a long time known to infest wild Poaceae plants in Kenya [12] until 2005 when it was first reported on maize [*Zea mays* L.] in Kakamega and Kisii [13]. During the first recovery, authors thought that *B. segeta* eggs may have been accidentally oviposited by gravid moths from wild hosts growing in the adjacent forests. Since then, this species has persisted in maize and sorghum [*Sorghum bicolor* (L.) Moench] fields in Kakamega, where sometimes its population exceeds *B. fusca -* the native dominant pest species [14]. Despite this growing importance, *B. segeta* infests a wide range of non-cereal graminaceous plants in the vicinity, making it the only native stem borer species found in both wild and cultivated fields in this area [15]. Host use diversification remains important to agricultural entomologists as it may mark the beginning of an evolutionary process that would result in a new pest. In addition to evolution of a new pest, host use diversification may sometimes result in adaptation to local conditions, or reduction in gene flow among populations using different hosts [16,17]. In this context, the source-sink role of cultivated and natural habitats in the life cycle and genetic composition of *B*. *segeta* has not been studied. Consequently, potential host use specialization and the reciprocal influence of populations from different habitats through exchange of individuals/genes are not known. 

The study of dispersal processes is a central problem in ecology, population genetics and conservation. For this reason, the estimation of dispersal rates has been one of the most investigated problems in population biology. Dispersal parameters can be directly estimated using ecological approaches such as mark-release-recapture methods but might not be applicable in studies involving sampling the propagules. In these cases, population genetics approaches provide a better alternative because the information contained in DNA can provide gene flow parameter estimates for different and complementary timescales [18,19]. Among molecular markers, mitochondrial markers, particularly the cytochrome *b* (Cyt. *b*) gene, has been applied in both phylogeographic studies [20] and estimation of long-standing historical host use differentiation [21]. In this study, the Cyt. *b* gene was used to examine host use diversification among *B*. *segeta* moths collected in the agricultural landscape along the Kakamega forest.

## 2. Experimental

### 2.1. Description of the Study Area

Kakamega Forest is located in western Kenya about 40 km North West of Lake Victoria. It is the only remnant of Guineo-Congolian rainforest in Kenya and was gazetted in 1933 by the Forest Department to enhance its protection [22,23,24]. Despite the conservation measures, high human population density in the area (175 individuals/km^2^) has led to considerable long-term human influence on the forest and its environs [24]. The area is generally suitable for rain fed agriculture (temperature ranges from 12.7 °C to 27.1 °C and average rainfall is 1,650 mm), and parts of the forest have been excised for establishment of crop fields as demand for agricultural land increases. Surveyed landscape, 21.2 km^2^ along the forest edge, was originally part of the main forest block but was opened for cultivation of maize and sorghum due to human population pressure [23]. It is thus characterised by cultivated fields interspersed with uncultivated areas.

The cultivated areas are covered by small farms, usually less than 2 ha, where maize, sorghum, finger millet and sugarcane are grown for domestic consumption. The uncultivated areas are covered by homes, forest edges and rivers. The natural habitats—hereafter referred to as wild habitats—support a wide range of plant species, some of which are potential hosts of stem borers [25]. The majority of the wild plants here that are considered as potential hosts of stem borers grow along the edge of the forest, around crop fields, and along the river banks. The area experiences a bimodal rainfall, which allows for two cropping seasons. The first season lasts from March to mid-July (long rain growing season, *LR*) and the second from mid-August to November (short rain growing season, *SR*). The area experiences light rain from the beginning of December to the end of February, hereafter referred to as non-cropping season.

### 2.2. Sampling, Rearing and Identification of Stem Borers

Surveys were carried out in both wild and cultivated habitats in 2005–2007 growing seasons (*SR*, 2005 & 2006; *LR*, 2006 & 2007). A total of 32 maize fields were identified and surveyed for stem borer infestation during each sampling session. In each field, 100 maize plants were randomly selected and inspected for stem borer infestation symptoms five weeks after germination. During field sampling, only infested stems were dissected for larval recovery. Since stem borer infestations are usually low in wild habitats [13,26], we adopted two sampling approaches during each sampling session. In the first approach, sampling was standardised with two researchers spending two hours inspecting plants belonging to Poaceae, Cyperaceae and Typhaceae families in natural habitats within 20 m of each cultivated field for infestation symptoms. In the second approach, researchers extended stem borer inspection beyond the field boundaries to capture populations that may lie beyond the 20 m field margin. Stem borer inspection was also extended to river banks and swamps. In each sampling session, all plant species belonging to Poaceae, Cyperaceae and Typhaceae families were carefully inspected for stem borer infestation symptoms or damage (scarified leaves, dry leaves and shoots, frass, dead hearts, holes bored). Infested plants were cut and dissected in the field for recovery of larvae and pupae. GPS of points from which stem borer larvae and pupae were recovered was recorded for distance based analysis.

Larvae recovered from both cultivated crops and wild hosts were reared on artificial diet according to the method described by Onyango and Ochieng-Odero [27]. Upon pupation, the materials were removed from the diet and kept in separate plastic vials where they were maintained until emergence. Emerging moths were identified using external morphological features (wing pattern, antennal type and genitalia). Voucher specimens were deposited in the Muséum National d'Histoire Naturelle (MNHN, Paris, France) and in ICIPE Biosystematics Unit—BSU (Nairobi, Kenya). The identified *B*. *segeta* moths were preserved in absolute ethanol to allow for further investigation on possible population exchange between wild and cultivated crops, and carry-over between growing seasons.

### 2.3. DNA Extraction and Sequence Analysis

Total genomic DNA was extracted from the thoracic muscles using a commercial kit (DNeasy^TM^ Tissue Kit, Qiagen GmbH, Germany) with Proteinase K digestion as recommended for animal tissues. The extracted DNA was stored at −20 °C until required for amplification. Polymerase chain reaction (PCR) was used to amplify a 709 bp Cyt. *b* fragment using the primers CP1 (5'-GATGATGAAATTTTGGATC-3') (modified from Harry *et al* [28]) and Tser (5'-TATTTCTTTATTATGTTTTCAAAAC-3') [29]. The PCR was performed on a Biometra GeneAmp PCR System in a 25 μL reaction mixture containing 1 μL of the genomic DNA, 1X Green GoTaq® Flexi Buffer, 0.24 mM dNTPs, 3 mM MgCl_2_, 0.4 µM of each primer and 1 unit of Taq polymerase (GoTaq®, Promega). After initial denaturation at 94 °C for 5 min, PCR condition was 40 cycles of 94 °C for 1 min of denaturation, 46 °C for 1 min 30 s of annealing, 72 °C for 1 min 30 s of extension and a final extension period of 10 min at 72 °C. The PCR products were visualised by means of electrophoresis in 1% agarose gel previously stained with ethidium bromide before UV exposure to verify amplification. Amplified products were purified with the Promega Wizard SV Gel and PCR Clean up System following the manufacturer’s protocol. DNA sequencing reactions were performed using the ABI PRISM® BigDye™ Terminator v3.0 Ready Reaction Cycle Sequencing Kit (Applied Biosystems), cleaned using ethanol/EDTA precipitation. Sequences were visualized on an ABI 3130 automated sequencer using Big-Dye fluorescent terminators. The consensus sequences were obtained after aligning respective forward and reverse sequences manually using Mac Clade 4.05 [30]. Consensus sequences were deposited in the Genebank (Accession numbers EU526412-EU526556). Sequences of other noctuids [*Busseola fusca* KI, *Busseola fusca* KII, *Sesamia calamistis* (*Panicum maximum*), *Sesamia calamistis* (*Zea mays*), *Manga nubifera*, *Manga melanondonta*] were obtained from the GeneBank to confirm *B. segeta* identity and determine its genetic distance relative to other species. Two mitotypes of *B. fusca*, named KI and KII above, co-exist in Kenya with individuals of KII more widely distributed than those of KI [20].

### 2.4. Data Management and Statistical Analysis

Sequences of individuals from different host plant species were grouped with respect to habitats (wild and cultivated) and seasons (*LR* and *SR*) for estimation of genetic diversities and rates of population exchange. Basic sequence statistics were calculated using DnaSP [31] and haplotype parsimony networks drawn using TCS 1.21 software [32]. The following parameters were used to estimate genetic variability among populations in wild and cultivated habitats, and among *LR* and *SR* growing seasons: number of haplotypes (*h*), number of polymorphic sites (*S*), haplotype diversity (*Hd*) [33], nucleotide diversity (*Pi*) [34] using the Jukes and Cantor correction [35], mean number of nucleotide differences (*K*) [36]. The extent of genetic differentiation between the populations (*F*_ST_) [37] was performed with the Arlequin ver. 2.0 Software [38]. Analysis of molecular variance (AMOVA) was used in Arlequin to indirectly assess the exchange and carryover of stem borer populations between habitats (cultivated fields/wild habitats) by comparing differences in haplotype composition in different habitats according to the growing seasons (LR/SR).

## 3. Results

### 3.1. Species Identity and Host Use Diversity

*Busseola segeta* can sometimes be confused for *B. fusca* when identification is done either at early instars of the larval stage or using wing patterns at the adult stage. Genetically, this species is different from other species in the region and there are limited chances of misidentification (Table 1). Closest species to *B. segeta* are the *B. fusca* clades (*KII* and *KI*) which are about 7.7 and 8.4% different, respectively. The other species, *S. calamistis*, *M. melanodonta* and *M. nubifera*, are more than 10% different.

*Busseola segeta* larvae were recovered from a total of nine different plant species, belonging to the Panicoideae grass sub-family. Infested grasses included *Cymbopogon nardus*, *Euclaena mexicana*, *Panicum maximum*, *Pennisetum macrourum*, *Pennisetum purpureum*, *Pennisetum unisetum*, *Saccharum officinarum*, *Sorghum bicolor* and *Zea mays* (Table 2). Apart from maize, sorghum and *P*. *purpureum*, which occupied large areas in the cultivated habitats [39], the other infested plants were localised in distribution and occurred mainly in less disturbed patches along the edges of cultivated fields and riverines.

**Table 1:** Genetic distance between *B. segeta* and other known stem borer species from Kenya. Cytochrome *b* sequences of the other species were downloaded from the GeneBank.

Stem borer species	*B. fusca* (KII)	*B. fusca* (KI)	*S. calamistis* (Crop)	*S. calamistis* (Wild)	*M. melanondota*	*M. nubifera*	*B. segeta*
*B. fusca* KII)	–	–	–	–	–	–	–
*B. fusca* (KI)	0.026	–	–	–	–	–	–
*S. calamistis* (Crop)	0.131	0.127	–	–	–	–	–
*S. calamistis* (Wild)	0.122	0.118	0.019	–	–	–	–
*M. melanondota*	0.133	0.124	0.133	0.138	–	–	–
*M. nubifera*	0.134	0.122	0.143	0.136	0.066	–	–
*B. segeta*	0.077	0.084	0.135	0.133	0.124	0.133	–

Note: The above species belong to three different genera: *B* (*Busseola*), *S* (*Sesamia*) and *M* (*Manga*).

**Table 2:** Plant species infested by *Busseola segeta* in the surveyed agricultural landscape in Kakamega during long and short rain growing seasons. Asterisks (*) indicate the cultivated host plants.

Host plant species	Total number of larvae recovered	
	*Long rain season*	*Short rain season*
*Cymbopogon nardus *(L.) Rendle	1	-
*Euclaena mexicana *Schrader	2	-
*Panicum maximum* Jacquin	28	29
*Pennisetum macrourum* Trinius	9	1
*Pennisetum purpureum* Schumach*	18	25
*Pennisetum unisetum *(Nees) Benth.	-	12
*Saccharum officinarum* L.*	2	2
*Sorghum bicolor *Delile*	18	-
*Zea mays *L*	65	115

### 3.2. Genetic Diversity and Differentiation in Host Utilization

**Figure 1:**
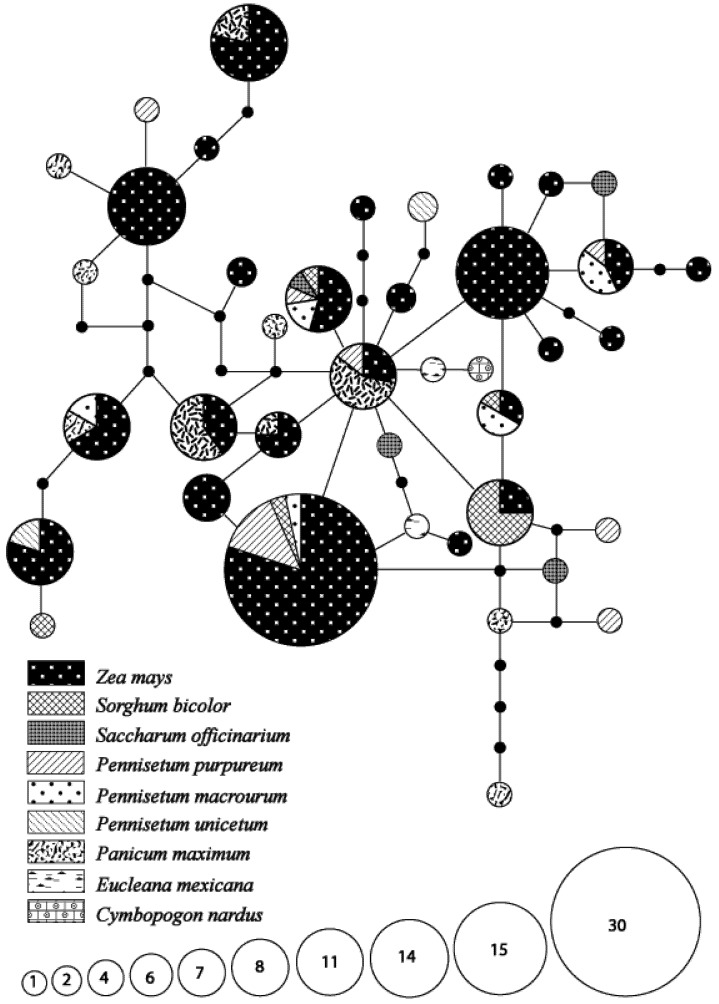
TCS mitochondrial haplotype network of *Busseola segeta* individuals collected from different host plants in Kakamega. The area of each circle is proportional to the number of samples in each haplotype. Lines represent single nucleotide mutations and black circles represent haplotypes that are not observed in the sample. Different shading patterns represent the different sampled host plants.

TCS parsimony network (0.95 parsimony limit) built from 147 *B*. *segeta* sequences revealed 40 haplotypes (*h*) with slight variation in distribution among the habitats (Figure 1). Twenty-eight of these haplotypes were found from collections made on maize, sorghum and sugar cane and 23 from wild host plants (see summary in Table 2). Ten out of all collected haplotypes were found in both wild and cultivated hosts. Despite the high number of haplotypes in the cultivated habitat (*h *= 28) compared to wild host plants (*h *= 23), the latter had relatively higher average number of nucleotide differences (K) and haplotype diversity (*Hd*) (Table 3). This partly explains the observed differentiation between the two habitats (*F_ST_*= 0.016; *p *= 0.015).

### 3.3. Seasonal Variations in Haplotype Composition

**Figure 2:**
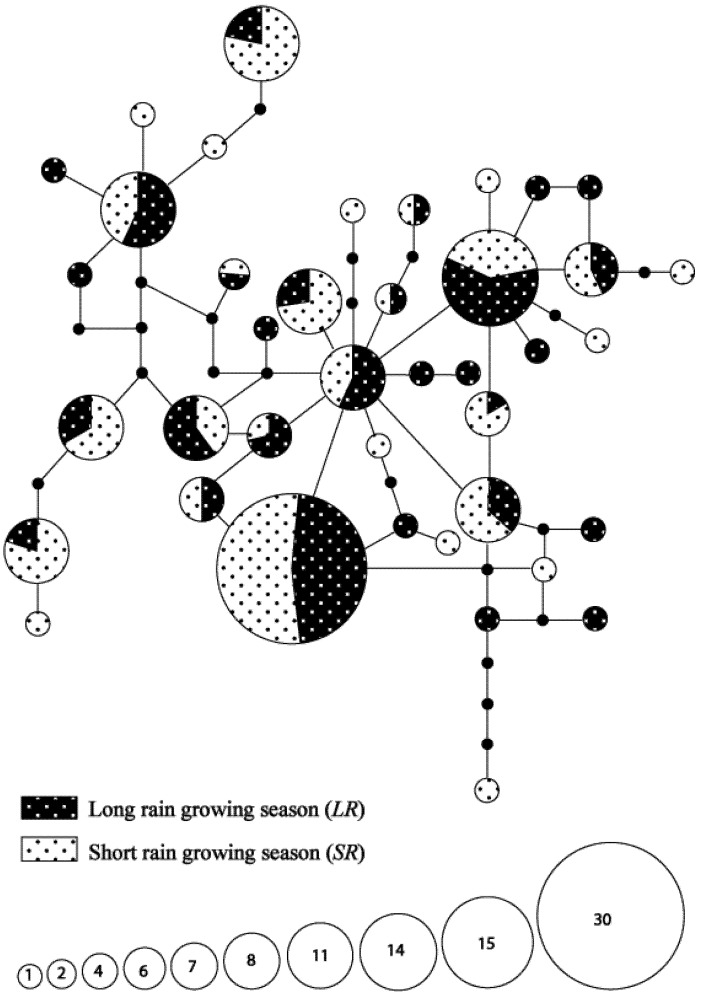
TCS mitochondrial haplotype network of *Busseola segeta* individuals collected during different seasons. Different shading patterns represent the different seasons. The area of each circle is proportional to the number of samples in each haplotype. Lines represent single nucleotide mutations and black circles represent haplotypes that are not observed in the sample.

A total of 28 haplotypes were found during the *SR* season of which 20 were found on cultivated host plants and 19 on wild host plants (see Table 2 and Figure 2). Though only 10 haplotypes were common to both habitats, there was no evidence of variation in genetic composition between the wild and cultivated habitats (*F*_ST_ = 0.017; *p *= 0.102). Similarly, non-significant variation in haplotype composition was observed in the *LR* season despite variations in haplotype numbers between the two habitats (*F*_ST_ = 0.019; *p *= 0.092). However, there was evidence of variation in genetic composition between growing seasons in the wild habitat (*F*_ST_ = 0.060; *p *< 0.001), with more haplotypes found during the *SR* season. Out of the 25 haplotypes identified from wild host plants, only 10 were common to both seasons, the majority of which were sampled from *P*. *purpureum*.

### 3.4. Hypothetical Exchange of Haplotypes between Habitats and Seasons

**Figure 3:**
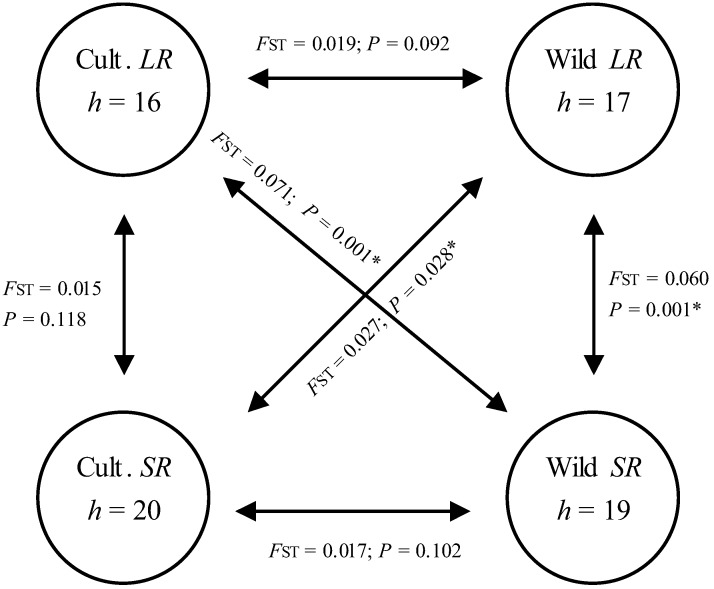
Summary of assumed exchange of haplotypes between habitats and seasons (indicated by arrows). Each unit is assumed to receive and give immigrants to each of the three units. *h* represents the number of haplotypes found in each unit while *F*_ST_ and *P* values are the AMOVA results computed between respective units. Asterisks (*) indicate where haplotype compositions between respective units varied significantly (*p* < 0.05).

The two habitats and two growing seasons (*LR* and *SR*) may be considered theoretically as four independent units (cultivated habitat *LR* and *SR*, and wild habitat *LR* and *SR*) within which there is continuous exchange of moths. Haplotypes found in any of the four units at a given time of the season can therefore be considered as products of either carry-over or movement of moths from at least one of the three units as summarised in Figure 3. This summary is limited to comparison of haplotyes in the above four units ignoring the possible influence of immigrant populations from the forest. Results reveal the existence of free exchange of haplotypes between seasons and habitats except in isolated cases where there was evidence of variation in haplotype composition. Significant variation was observed between wild *SR* against both wild *LR* (*F*_ST_ = 0.060; *p *< 0.001) and cultivated *LR* (*F*_ST_ = 0.071; *p *< 0.001). Variation was also observed between wild *LR* and cultivated *SR* (*F*_ST_ = 0.027; *p *= 0.028) despite the high number of haplotypes (12) shared between the units. 

**Table 3:** Genetic diversity of the Cytochrome *b *gene in *Busseola segeta* populations from different hosts across the seasons.

Genetic parameters		Cultivated host plants			Wild host plants		
		LR	SR	Total	LR	SR	Total
Number of sequences		42	36	78	35	34	69
Number of segregating sites, *S*		20	28	35	25	20	28
Number of haplotypes, *h*		16	20	28	17	19	23
Haplotype diversity, *Hd*		0.904	0.948	0.932	0.946	0.950	0.951
Average number of differences, *K*		3.113	3.632	3.375	3.832	3.403	3.641
Nucleotide diversity, *Pi*		0.004	0.005	0.005	0.005	0.005	0.005
AMOVA results	*F_ST_*	0.015			0.060		
	p	0.118			0.001		

## 4. Discussion

Changes in land use pattern are thought to be among the factors that accelerate evolution of insect pests [40,41]. Despite the varied changes in land use practice in Kakamega during the last decades [24], scientists never imagined that *B*. *segeta* could become an important pest of cereal crops [13]. This study confirms establishment of this species in cereal crops along the edge of the Guineo-congolian rain forest relict in Kakamega. However, this is not the first time an indigenous stem borer species expanded its diet breadth to include cultivated crops [10]. *Eldana saccharina* is the most recent species that initially colonized mainly sedges and expanded its diet breadth to include sugarcane in both western and southern Africa countries [42].

Several theories have been brought forward to explain host use diversification among phytophagous insects. One theory, as proposed by Futuyma [43], argues that diversity of plants—particularly their chemical diversity—could be involved in host use diversification. However, according to what could be called the ‘explosive adaptive radiation theory’, a "key" character evolves in a lineage that enables it to explore new niches. Associated with habitat modifications, the availability of new resources as well as new areas to colonize could promote speciation processes (see Futuyma [44]). An example could be the chemical defenses of plants, which are often thought to be limiting for exploitation of the plants by insects [45]. As soon as an insect has been able to overcome that defence, an opportunity for diversification can occur [45,46]. Unlike the specialized stem borer species *B. fusca*, which is found on limited host plants, *B. segeta* larvae were found on a wide range of hosts in both wild and cultivated habitats. Though not tested, its presence on a wide range of hosts may be attributed to its inherent potential to overcome plant defence without undergoing genetic adaptation. This is contrary to the observed long historical host use adaptation reported on *S. calamistis* in Kenya [47]. The ability to overcome plant defence allowed free movement of its moths between habitats as confirmed by the general lack of genetic structure among larvae found in different habitats and growing seasons. Unlike *B. fusca* [13] or *S. calamistis* [47] that show host use adaptation, *B. segeta* is an example of a phytophagous insect that utilizes plants with human induced distribution range, maize, but do not show evidence of host race formation or reduction of gene flow among populations using different hosts.

The movement of *B*. *segeta* moths between host plants in different habitats and subsequent persistence may be attributed to host use plasticity. In the "plasticity theory", phytophagous insects are thought to be carrying genotypes for plasticity that allow them to broaden their resource use when new resources become available [48,49]. Janz *et al.* [50] used this theory to explain the increased likelihood of nymphalid butterfly tribe Nymphalini colonizing ancestral host *Urtica dioica* or related plants during their study. Though *B. segeta* moths appear to fly long distances, their host use plasticity can function as pre-adaptation to novel environments, an attribute that dictates response of many phytophagous insects [51,52]. *Busseola segeta* larvae were found mainly on plants belonging to subfamily Panicoideae with higher infestation on maize and *P. maximum*. Like other phytophagous insects [9], *B. segeta* appears to have kept a chemical "memory" of some plants belonging to subfamily Panicoideae used earlier in the history of their lineage. They may therefore have pre-adapted to utilize any representative of this subfamily not presently used by the females for oviposition. However, this mechanism may not apply to all stem borers since some species like *B*. *fusca* have specialized and currently use limited number of hosts [13,14].

Management of stem borer pests has been one of the priority areas among agricultural entomologists in Africa [13,53,54] and the addition of an extra species to the pest community would complicate the existing management practices. Therefore, movement and subsequent establishment of *B*. *segeta* as a maize pest is of great concern to entomologists [14]. Though *B*. *segeta* is currently seen as a less important pest due to the general low stem borer infestation levels in Kakamega area, it is one example of a poorly known stem borer species that could gradually become an important pest. In addition to *B. segeta*, there are many other stem borer species in wild habitats that are known to act as alternative hosts of natural enemies [25,55]. However, as demand for more agricultural land increases, the non-cultivated fragments are likely to be cleared, affecting their role both as reservoirs of stem borers and as refuges for natural enemies, making it difficult to reverse conditions that drive host use expansion. There is therefore the need to protect natural habitats and uncultivated fragments around crop fields to maintain the species diversity and provision of ecosystem services.

## 5. Conclusion

This study confirms establishment of *B. segeta* in crop fields around the Kakamega forest. Though the density of this moth is lower than economically important pest species in the area [25], the *B. segeta* moths did not exhibit genetic variation in terms of host use. *Busseola segeta* presence in a wide range of hosts without genetic variation strongly suggests the existence of host use plasticity. This points to its ability to overcome plant defence without undergoing genetic adaptation. *Busseola segeta* is thus capable of becoming an important pest in the region and control measures need to be put in place to contain its potential distribution.
